# Buspirone in the management of refractory irritable bowel syndrome: A case report

**DOI:** 10.1097/MD.0000000000028003

**Published:** 2021-12-23

**Authors:** Mustafa Abdul Karim, Nadeen Al-Baz, Peter M. Haddad, Shuja M. Reagu, Majid Alabdulla

**Affiliations:** a Department of Psychiatry, Hamad Medical Corporation, Doha, Qatar; b Weill Cornell Medicine, Qatar; c Department of Internal Medicine, Hamad Medical Corporation, Doha, Qatar; d College of Medicine, Qatar University, Doha, Qatar.

**Keywords:** abdominal pain, buspirone, irritable bowel syndrome

## Abstract

**Rationale:**

Irritable bowel syndrome (IBS) is a chronic and debilitating functional disorder of the gastrointestinal tract manifested by abdominal pain and bowel habit dysregulation. The pathophysiology is complex and management targets symptom resolution. Therapeutic interventions range from dietary modification, psychological interventions, exercise, to the use of antispasmodics, antibiotics, and antidepressants. Anecdotal reports have suggested that buspirone may be beneficial in the treatment of functional dyspepsia and IBS and its physiological effect of reducing gastric tone provides a rational for its benefit.

**Patient concerns:**

A 28-year-old man with unremarkable past medical and psychiatric history presented with worsening abdominal pain, bloating, and bowel movement dysregulation of over 6-year duration.

**Diagnoses:**

Physical examination revealed mild distension and discomfort on deep palpation. Thorough blood investigations, stool analysis and culture, and imaging were unremarkable except for the detection of mucus with stool. The patient was diagnosed with irritable bowel syndrome with mixed habits.

**Interventions:**

Dietary adjustment and a range of medications (mebeverine, simethicone, loperamide, rifaximin, sertraline and amitriptyline) yielded unsatisfactory response of were not tolerated. Buspirone was eventually introduced.

**Outcomes:**

Buspirone was associated with a significant and sustained improvement in IBS symptoms and quality of life.

**Lessons:**

This case suggests that buspirone was effective in treating refractory IBS. Further research is needed to assess the role of buspirone in IBS management.

## 1 Introduction

Irritable bowel syndrome (IBS) is a chronic and debilitating functional disorder of the gastrointestinal tract manifested by abdominal pain and bowel habit alterations of unclear etiology.^[1]^ It is one of the most common gastrointestinal diseases, affecting 5% to 20% of the general population.^[2]^ IBS is diagnosed more frequently in women and its prevalence decreases with age.^[3,4]^ Although not life-threatening, the disorder places a large economic burden on the healthcare system,^[5,6]^ impairs individuals’ work productivity,^[7]^ and quality of life.^[8]^ High covariance between IBS and mental disorders has additionally been reported.^[9,10]^

The pathophysiology of IBS is multipronged and uncertain, including gastrointestinal motility dysregulation, visceral hypersensitivity, bacterial overgrowth, intestinal inflammation, food sensitivity, psychological factors, and brain–gut interaction.^[11,12]^ It is a functional disorder of set symptoms in the absence of clear organic etiology and diagnosis hence relies on careful history taking, physical examination, and relevant investigations. The Rome IV definition of IBS requires the presence of abdominal pain at least 1 day on average per week in the last 3 months associated with ≥2 of the following: related to defecation; associated with a change in frequency of stool; associated with a change in stool form.^[13]^ In addition, criteria must be fulfilled for the last 3 months with symptom onset at least 6 months before diagnosis.^[14]^ The disorder is classified based on the patient's perceived stool consistency rather than the frequency of defecation. Stool consistency can be assessed using the Bristol stool form scale,^[15]^ and IBS is categorized into 4 subtypes based on the proportion of symptomatic movements. IBS with predominant constipation is diagnosed when the patient reports more than a quarter of bowel movements with Bristol stool form types (BSFT) 1–2 and less than a quarter with BSFT 6–7; IBS with predominant diarrhea (IBS-D) with over a quarter of bowel movements with BSFT 6–7 and less than a quarter with BSFT 1–2; IBS with mixed habits (IBS-M) involves over a quarter of bowel movements with BSFT 1–2 and over a quarter with BSFT 6–7; Unsubtyped IBS for patients who meet diagnostic criteria for IBS but whose bowel habits cannot be clearly defined or categorized.^[14,16]^

Management is multifaceted and targeted towards symptom resolution. The National Institute of Health and Care Excellence recommends more frequent meals of smaller proportions and avoidance of trigger foods, caffeine, and excess alcohol.^[17]^ Eswaran et al's randomized-controlled trial found adequate symptom relief with low in fermentable oligosaccharides, disaccharides, monosaccharides and polyols diet (LFD) in 40% to 50% of patients with IBS-D or diet based on modified National Institute of Health and Care Excellence guidelines, with the former yielding significantly higher proportion of abdominal pain responders.^[18]^ Lifestyle measures such as regular exercise^[19]^ and adequate sleep^[20]^ can offer further symptomatic relief.

Antispasmodics offer modest improvement, and antidepressants including serotonin-selective reuptake inhibitors (SSRIs) and tricyclic antidepressants (TCAs) have shown to be effective, although results of different trials varied based on patient characteristics.^[21]^ A more recent review discussed the potential benefit of TCAs in IBS-D and SSRIs for irritable bowel syndrome with predominant constipation or IBS associated with psychological comorbidities.^[22]^ We present a case of a young man with IBS. Following several unsuccessful treatment trials, he reported significant improvement with buspirone.

## 2 Methods

### 2.1 Ethics approval and consent to participate

The patient provided signed consent for participation and publication of this article. The proposal was accepted by the international review board (IRB) at Hamad Medical Corporation (Protocol ID: MRC-04–21–846).

### 2.2 Case presentation

The patient is a 28-years-old Middle Eastern man who attended the primary healthcare clinic reporting bloating, abdominal pain, and dysregulated bowel habits of several years’ duration. He began experiencing symptoms at the age of 22 with gradual progression to present date. Abdominal pain and bloating were experienced most days of the week, and pain varied in severity ranging from 4 to 8/10, with 10 being the worst pain ever experienced. Abdominal bloating and pain were mainly postprandial, and not restricted to the intake of lactose-containing products. Aggravating factors included stress, large-portion meals, and spicy food. High-intensity exercise such as running or playing soccer worsened abdominal pain and bloating and the patient hence preferred walking or resistance training. Pain and bloating temporarily subsided following defecation. Bowel habits ranged from 3 to 6 watery or mushy bowel movements during the day, alternating with periods of constipation consisting of hard and lumpy bowel motions only once or twice a week. At the time of first presentation, the patient was experiencing more than a quarter of bowel movements with watery stool consistency. He occasionally experienced mild nausea not associated with vomiting and did not notice significant weight change since the onset of symptoms. Symptoms were not associated with bloody or black stools, tenesmus, fecal urgency, or heartburn. Review of systems was otherwise unremarkable.

Physical examination in the primary healthcare clinic revealed mild distention, discomfort with deep palpation, and hyperactive bowel sounds. Full blood count and comprehensive metabolic panel including electrolytes and minerals, liver and kidney function tests, amylase and lipase levels, erythrocyte sedimentation rate, and C-reactive protein were within reference range. Tests for thyroid function test and serum endomysial antibody did not show any abnormalities. Stool analysis revealed the presence of mucus but no ova or parasites. Stool cultures were unremarkable. The patient was provisionally diagnosed with IBS, advised to avoid triggering food items, and commenced on Mebeverine 200 mg modified release capsules twice daily (BID) in addition to Simethicone 125 mg up to 3 times daily (TID) as needed after meals for bloating. On follow-up the patient reported mild improvement in flatulence but persistent abdominal pain and no change in bowel movement dysregulation.

The patient was referred to internal medicine and gastroenterology clinics for further investigation and management. Comprehensive history taking revealed that he was unable to precisely recall the duration of periods during which he experienced constipation versus diarrhea/loose stool, but estimated over a quarter of bowel movements with BSFT 1–2 (separate hard lumps, or lumpy and sausage like) and over a quarter with BSFT 6–7 (mushy or liquid consistency). Urea breath test, *Helicobacter pylori* stool antigen, fecal occult blood test, a second stool analysis and culture, upper and lower endoscopy revealed no abnormalities. The gastroenterologist diagnosed the patient with IBS-M advised him to follow the LFD. Mebeverine and simethicone were discontinued and loperamide up to 16 mg/d introduced as he suffered predominantly from loose stools and diarrhea on follow-up. In addition, he was prescribed a 2-week course of rifaximin 550 mg TID. He did not request sick notes or a medical report recommending an amendment in occupational responsibility and his main concern was symptom resolution. He later presented to the emergency department complaining of severe abdominal pain, bloating, and constipation. He had been adherent to the LFD 4–5 days per week, and symptoms were not associated with fever, nausea, vomiting, black or tarry stools, or rectal bleeding. He reported feeling anxious lately due to recent marital conflicts and increased work-related stress. Review of systems and physical examination were unrevealing except for abdominal distention and discomfort on deep palpation. Abdominal X-ray showed fecal buildup and CT abdomen revealed no structural abnormalities. Liquid-based enema and laxatives helped relieve the constipation and abdominal symptoms. The consultation-liaison (CL) psychiatry team was consulted to address his anxiety symptoms. The patient reported feeling anxious and easily irritable, had poor concentration and felt tired during the day. He attributed his psychological distress to his abdominal symptoms as they led to absences or missing deadlines at work and marital conflict. The latter arose as he preferred to stay home and where possible avoided social activities for fear of unpredicted exacerbation of abdominal symptoms.

The CL team discussed several management options to address the patient's anxiety symptoms and the possible differential diagnosis of somatic symptom disorder. The patient was reluctant to start psychotherapy due to time constraint, but consented to a trial of sertraline 25 mg daily. He reported worsening abdominal pain, nausea, heartburn, and watery diarrhea on follow-up. Despite taking sertraline after meals and in different times of the day (morning or afternoon), his abdominal symptoms continued to worsen. He discontinued sertraline after 6 days, and now reported that his bowel movements changed from constipation to frequent loose-motion. Sertraline was discontinued and he was started on amitriptyline 10 mg at bedtime. He reported no noticeable effect after 3 weeks. When the dose was increased to 25 mg at bedtime, the patient reported improved sleep quality and felt calmer during the day. Frequency of bowel movements decreased, but abdominal pain and bloating persisted. Doses of amitriptyline 35 to 50 mg caused dizziness during the morning, slowing of bowel movements to 1 or 2 movements per week, associated with increased abdominal pain and bloating.

**Figure 1:**
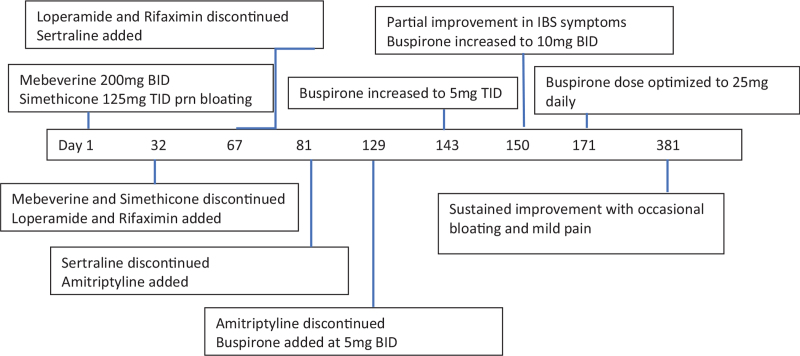
Timeline of events.

The patient discontinued amitriptyline due to worsening constipation. He was initially reluctant to explore further psychotropic options, but later agreed for a trial of buspirone. He experienced no benefit at a dose of 5 mg BID. At 5 mg TID, he started experiencing partial improvement in abdominal pain and bloating. When the dose was increased to 10 mg BID, he initially experienced morning dizziness which spontaneously resided after 2 weeks. Bloating and abdominal pain significantly improved. He was passing stool 2 to 3 times per day, and stool consistency was occasionally loose. Increasing the dose to 10 mg TID caused excessive dizziness and the dose was finally tailored to 10 mg in the morning, 5 mg in the afternoon, and 10 mg at bedtime. He reported good tolerability to buspirone, sustained improvement in symptoms, and a consistent pattern of passing 2 to 3 bowel movements daily with BSFT 3–5. Gastrointestinal symptom relief enabled him to function better at work. He was now able to concentrate more at the task at hand rather than constantly worrying about and being distracted by his symptoms. In addition, he felt less tense and anxious, and started joining outings more frequently with his wife and friends. Although he continued to experience occasional bloating and mild pain, symptoms were tolerable and he was satisfied with the overall response from buspirone. At the time of writing he has been taking buspirone for a total of 7 months and his IBS symptoms have been relatively well controlled throughout. This is the longest period without significant gastrointestinal (GI) symptoms that he has experienced since his IBS started 6 years previously (Fig. 1).

## 3 Discussion

The patient we report gave a history of abdominal pain, bloating, bowel movement dysregulation, associated with occasional mild nausea. Abdominal pain and bloating were most prominent after food intake, and symptoms were aggravated by stress, large-meal and spicy food intake, and high intensity exercise. Temporary relief of symptoms was experienced after bowel movements. Physical examination and investigations were unremarkable except for mild distension and discomfort with deep palpation, occasional hyperactive bowel sounds, and presence of mucus with stool. Although nonspecific, Thompson et al^[23]^ reported that a sensation of incomplete evacuation and mucus discharge with bowel movements can support the diagnosis of IBS. The most likely diagnosis was IBS-M based on the ROME IV criteria.^[24]^

Differential diagnoses for organic causes were considered but excluded. Peptic ulcer disease can present with abdominal pain and fullness, nausea, and vomiting. However, blood investigations, urea breath test, *H pylori* stool antigen test, and endoscopy did not show any abnormalities. Gastroenteritis is also unlikely as the patient was afebrile, and physical examination was unrevealing with no leukocytosis or elevated inflammatory markers. Although stool analysis showed the presence of mucus, no ova or parasites were detected and stool cultures were unremarkable. The absence of derangements on full blood count, comprehensive metabolic panel, and thyroid function tests, and negative endomysial antibody test rendered hypercalcemia, hyper- or hypothyroidism, celiac disease, and pancreatitis unlikely etiologies. Lactose intolerance was less likely as symptoms did not specifically emerge after the intake of lactose-containing products and persisted after following the LFD. Hydrogen breath test or lactose tolerance tests were not done. The absence of blood in stool, and lack of abnormalities on upper and lower endoscopy, abdominal X-ray, and CT abdomen makes it unlikely that the patient suffered from inflammatory bowel disease, chronic mesenteric ischemia, or oncologic etiology.^[25]^

The patient reported distressing symptoms affecting his quality of life and his main concern was symptomatic relief rather than worrying about having a serious illness. Illness anxiety disorder is hence unlikely. The patient spent significant amount of time worrying about his symptoms and changing his lifestyle and behavior in anticipation of symptom exacerbation. In addition, he was increasingly anxious about his symptoms as they progressed, and the differential of somatic symptom disorder cannot be ruled out. Differential diagnoses of malingering or factitious disorder are unlikely as there was no reason to suspect that the patient was intentionally falsifying or exaggerating his symptoms nor was there evident primary or secondary gain.^[26]^

Varying degrees of efficacy have been reported pertaining to the treatment options offered and tried. A systematic review found a favorable response among IBS patients on LFD, although several limitations including adherence and cost were highlighted.^[27]^ Darvish-Damavandi et al^[28]^ showed that Mebeverine was well tolerated although efficacy did not achieve statistical significance. A more recent placebo-controlled double blind randomized clinical trial involving patients with IBS-D showed that controlled-release Mebeverine 200 mg BID administered over 8 weeks had modest but significant improvement in reducing the number of bowel movements, severity of abdominal cramps, and improving IBS-related quality of life. Given its modest effect, however, the authors concluded that it is a not a good choice for patients with severe symptoms.^[28]^ There is accumulating evidence highlighting the significance of bacterial overgrowth in IBS pathophysiology, and the role of rifaximin as a safe antibiotic with low oral bioavailability and bacterial resistance in improving IBS symptoms. Rifaximin is indicated in the United States and Canada for IBS-D treatment, and exerts its therapeutic effect through multifactorial mechanisms including gut microbiota manipulation, modulating inflammatory cytokines, and intestinal permeability.^[29]^ Although our patient was reluctant to engage in psychotherapy, evidence suggests significant and durable effect of cognitive–behavioral therapy on IBS symptoms and quality of life.^[30]^

Previous trials have shown a positive outcome of SSRIs in improving IBS symptoms and overall quality of life independent of their effect on mood symptoms.^[31]^ Conversely, Lin et al's population-based retrospective observational study found an increased adjusted hazard ratio of BIS in patients with anxiety disorders and individuals who were prescribed SSRIs.^[32]^ Furthermore, a recent analysis of 18 randomized clinical trials found a favorable outcome for TCAs, but not SSRIs in addressing IBS symptoms. Limitations such as lower placebo rates, data analysis and reporting, and publication bias must be taken into consideration when interpreting these findings.^[32]^

Buspirone is an azaspirodecanedione agent, originally developed as an antipsychotic but found to have a poor effect in treating psychosis. It exhibits its therapeutic effect mainly as a partial agonist at the post-synaptic serotonin type 1A (5-HT_1A_) receptors, and is additionally a weak antagonist at presynaptic dopamine D2, D3, and D4 receptors, and partial agonist at alpha-1 noradrenergic receptors.^[33]^ It was found to have anxiolytic properties and has recently emerged as a treatment option for generalized anxiety disorder with favorable side-effect profile.^[34]^ Several studies highlighted the role of buspirone in ameliorating symptoms of functional gastrointestinal disorders. Tack et al^[35]^ found that buspirone administration significantly reduced symptoms of overall dyspepsia, postprandial fullness, early satiety, bloating, and increased gastric accommodation. Further reports shed light on the buspirone's role in the management of functional dyspepsia.^[36]^ Oudenhove et al^[37]^ found a dose-dependent effect of buspirone in relaxing the proximal stomach and decreasing gastric emptying. Alternatively, a randomized double blind placebo controlled trial found no significant effect with buspirone administration in altering colonic compliance, tone, or sensation compared with placebo.^[38]^

The pharmacodynamic underpinnings for buspirone's positive effect in our case are speculative. 5-HT1 receptor agonists have been shown to display a potent analgesic effect in colorectal distension-induced visceral pain model in rats.^[39,40]^ In the human GI tract, the majority of serotonin, or 5-hydroxytryptamine (5-HT), is released from enterochromaffin cells and plays a pivotal role in regulating motor, sensory and secretory functions. In addition, it mediates the brain–gut connection through extrinsic innervation with the autonomic nervous system.^[41]^ 5-HT_1A_ receptors are mainly located in the myenteric and submucosal plexuses of the enteric nervous system and regulate the release of mediators including histamine,^[42]^ which is involved in a multitude of functions ranging from immunological and inflammatory regulation, the modulation of GI motility, nociception, gastric acid production, and mucosal ion secretion.^[43]^ Buspirone might have adjusted GI histaminergic release leading to bowel movement regulation and pain reduction. In addition, occupational stress has been shown to affect GI physiology by increasing visceral perception, altering GI motility and secretion, and increasing intestinal permeability.^[44]^ One of the main triggers in our case was work-related stress, and the anxiolytic properties of buspirone might have regulated the brain–gut axis and reduced the impact of stress on GI functioning.

To the best of our knowledge, this is the first case report on buspirone effectiveness in refractory IBS. The case highlights the need for a multidisciplinary approach in its management. Further research is needed to assess the role of buspirone in the management of IBS.

### 3.1 Strengths and limitations

The patient was diagnosed with IBS following thorough investigation rendering other organic causes unlikely. Diagnosis was also based on ROME-IV criteria. The management of this case followed international guidelines, and buspirone was only introduced after stepwise management failed to achieve a desirable response. Although the patient reported significant and sustained improvement with buspirone, utilizing IBS severity before and after the introduction of buspirone would have allowed for a more objective assessment of response.

## Acknowledgments

The authors thank the patient for the permission to report this case.

## Author contributions

Nadeen Al-Baz conducted the initial intake and follow-up for the patient's IBS presentation at the general medicine clinic and contributed to writing the manuscript draft. Mustafa Abdul Karim, Shuja Reagu, and Majid Alabdulla conducted the consultation and follow-up for the patient's mental health presentation. Peter Haddad contributed to case discussion and manuscript revision and completion. All authors read and approved the manuscript.

**Conceptualization:** Mustafa Abdul Karim, Shuja Reagu.

**Data curation:** Shuja Reagu.

**Investigation:** Mustafa Abdul Karim, Nadeen Al-Baz.

**Supervision:** Peter Haddad, Majid Alabdulla.

**Validation:** Peter Haddad.

**Writing – original draft:** Mustafa Abdul Karim, Nadeen Al-Baz.

**Writing – review & editing:** Mustafa Abdul Karim, Peter Haddad, Shuja Reagu, Majid Alabdulla.

## Correction

The disclosure “Open Access funding provided by the Qatar National Library” has been added to the footnote on the first page.
